# Exploration of the relationship between biogas production and microbial community under high salinity conditions

**DOI:** 10.1038/s41598-017-01298-y

**Published:** 2017-04-25

**Authors:** Shaojie Wang, Xiaocong Hou, Haijia Su

**Affiliations:** Beijing Advanced Innovation Center for Soft Matter Science and Engineering, Beijing Key Laboratory of Bioprocess, Beijing University of Chemical Technology, Beijing, 100029 People’s Republic of China

## Abstract

High salinity frequently causes inhibition and even failure in anaerobic digestion. To explore the impact of increasing NaCl concentrations on biogas production, and reveal the microbial community variations in response to high salinity stress, the Illumina high-throughput sequencing technology was employed. The results showed that a NaCl concentration of 20 g/L (H group) exhibited a similar level of VFAs and specific CO_2_ production rate with that in the blank group, thus indicating that the bacterial activity in acidogenesis might not be inhibited. However, the methanogenic activity in the H group was significantly affected compared with that in the blank group, causing a 42.2% decrease in CH_4_ production, a 37.12% reduction in the specific CH_4_ generation rate and a lower pH value. Illumina sequencing revealed that microbial communities between the blank and H groups were significantly different. *Bacteroides*, *Clostridium* and BA021 uncultured were the dominant species in the blank group while some halotolerant genera, such as *Thermovirga*, *Soehngenia* and *Actinomyces*, dominated and complemented the hydrolytic and acidogenetic abilities in the H group. Additionally, the most abundant archaeal species included *Methanosaeta*, *Methanolinea*, *Methanospirillum* and *Methanoculleus* in both groups, but hydrogenotrophic methanogens showed a lower resistance to high salinity than aceticlastic methanogens.

## Introduction

An increasing amount of food waste (FW) is produced by the sorting, cooking, peeling and dining processes^1^. According to the statistics, more than 2000 ton of FW is generated every day in Beijing^2^, and the high organics, salinity and water contents of FW have caused serious environmental problems in modern societies^3^. Within different possible treatment routes, anaerobic digestion (AD) of FW into biogas is proven to be an effective solution for FW treatment^4^. Although the composition of FW is highly variable depending on the collection sources, it usually contains high salinity. Oh *et al*. reported that the NaCl-amended FW contained 10 to 35 g/L NaCl, while the non-washed FW contained 11.6 g/L NaCl^5^. Dai *et al*. collected FW from cafeterias in Shanghai with NaCl concentration of 8.0 g/L^6^. Wang *et al*. found that the NaCl concentration from FW anaerobic digestate could reach 13.8 g/L^7^.

This high salinity could cause cell osmotic stress imbalance, resulting in plasmolysis and/or loss of activity of cells^8, 9^, which would further cause inhibition and even failure of the AD process. For instance, Nagai *et al*. found that the utilization of FW from soy sauce was difficult because of its high salinity of 10% (w/w) despite its highly nutritious biomass^10^. Lee *et al*. studied the effect of salinity on biogas production from food waste leachate, and found that 0.5~2 g/L NaCl would increase the methane yield while 5 and 10 g/L NaCl resulted in a reduction of methane yield by 36% and 41%, respectively^11^. Rinzema *et al*. found that the formation of methane from acetate would be inhibited by 10, 50 and 100% respectively at Na^+^ concentrations of 5, 10 and 14 g/L^12^.

Research on how high salinity would affect the biogas production has been drawing increasing attention. It was reported that a Na^+^ concentration ranging from 2 to 10 g/L would moderately inhibit the methanogenic activity, while a concentration exceeding 10 g/L would strongly inhibit methanogenesis^11, 13^. Lefebvre *et al*. reported that methanogenesis started to be affected at a NaCl concentration of 5 g/L while acidogenesis was severely affected only at NaCl concentration exceeding 20 g/L^8^. However, detailed and intensive analysis is still scarce. Exploring the influence of high salinity on biogas production in the AD process is therefore of great significance.

Generally, the AD process includes four steps, namely, hydrolysis, acidogenesis, acetogenesis, and methanogenesis^14^. The first three steps are mediated by bacterial populations in which organic matter is converted to volatile fatty acids (VFAs) and further digested into acetate, H_2_ and CO_2_. The last step is performed by the archaeal group that produces methane using the acidogenic products^14, 15^. The microorganisms in the four steps affect the production of methane in different ways. It is therefore important to comprehensively understand the microbial behavior to fundamentally improve the efficiency of the AD process. Although some researchers have investigated the influence of the high salinity inhibition on microbial community structure^8, 16^, few findings provide an entire and in-depth analysis by correlation of biogas production with microbial community structure.

The microbial community of biogas production is commonly determined via construction of 16S rRNA clone libraries and Denaturing Gradient Gel Electrophoresis (DGGE)^17^. However, it is too difficult to investigate a complex microbial community by DGGE due to the limited information and low resolution^18^. The analysis of the microbial community using a clone library is moreover very tedious and expensive^19^. With the ongoing development of sequencing technology, the high-throughput sequencing shows a high efficiency in microbial community structure identification. Especially, Illumina sequencing has been widely used for its low cost and high sequence depth merits^20^.

In this study, the culture-independent Illumina high-throughput sequencing technology was employed and the microbial community changes between the blank and high salinity groups (H group) were systematically investigated and compared. The objectives were: to evaluate the influence of increasing NaCl concentrations on the biogas production; to reveal the bacterial and archaeal communities diversity and structure; and to explore the relationship between microbial community structure and process performance under high salinity conditions.

## Results

### Effect of increasing NaCl concentrations on biogas production

Prior to this study, the effects of different substrate concentrations (6, 9, 12 g/L starch) on the biogas production were firstly investigated. The results showed that the methane yields were 305.91, 236.90 and 245.71 mL/g-VS, respectively (Table S1). Therefore, 6 g/L starch was chosen as the concentration for the subsequent experiment.

**Figure 1:**
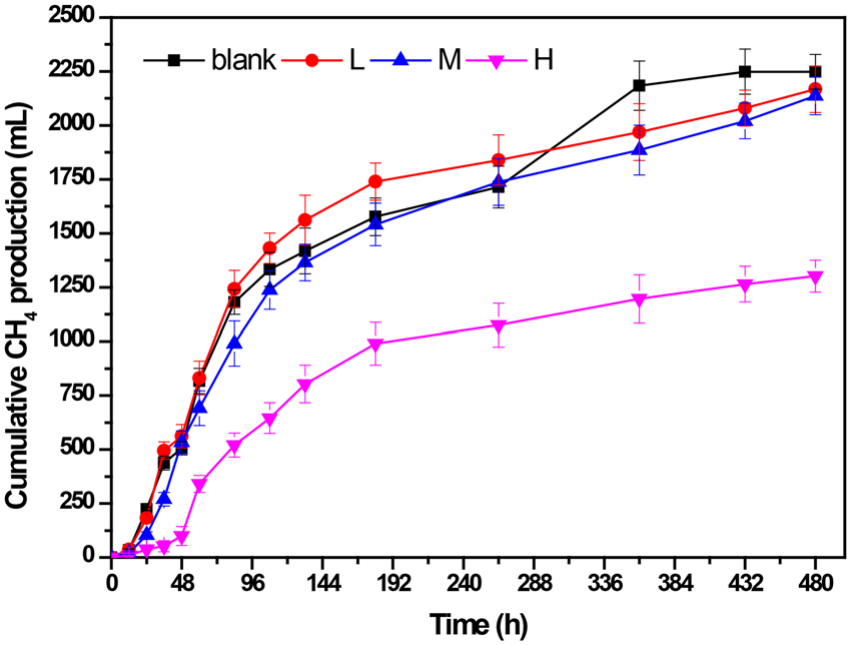
Cumulative methane productions at different concentrations of NaCl. Blank group (0 g/L), L group (5 g/L), M group (10 g/L) and H group (20 g/L). The experiments were operated in 1 L digesters at mesophilic temperature of 35 ± 1 °C.

To investigate the influence of high salinity conditions on the biogas production, a blank group (0 g/L NaCl) and three supplement groups (L, M, and H group) with increasing NaCl concentrations (5, 10 and 20 g/L NaCl respectively) were assessed. The effects of increasing NaCl concentrations on characteristics of different groups were shown in Fig. 1.

#### CH_4_ production

Generally, methane production rates of each group were higher in the early stage of fermentation (0~92 h), but gradually decreased thereafter (Fig. 1). The final cumulative CH_4_ productions for blank, L, and M group were 2249.0 ± 131.3 mL, 2167.3 ± 127.6 mL and 2137.2 ± 138.1 mL respectively, which were nearly identical with a slight decrease at increasing NaCl concentration. Notably, the supplement of 5 g/L NaCl (L group) could even promote the biogas production before 264 h, although an overall higher CH_4_ production was obtained at the end of fermentation in the blank group. The methane production in the H group was significantly reduced to 1301 ± 125.7 mL compared with other three groups (all *P* < 0.05), indicating that NaCl inhibition clearly occurred in the H group. Besides, a distinct lag phase (0~48 h) could also be observed in the H group.

#### pH

**Figure 2:**
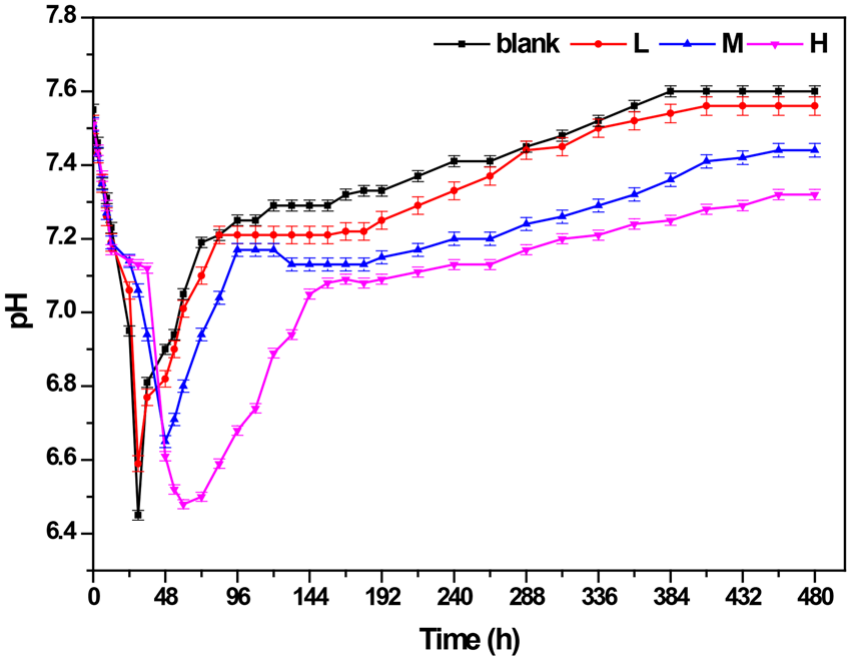
pH variation at different concentrations of NaCl.

As can be seen in Fig. 2, the addition of NaCl differently influenced the pH value. The pH value in both the blank and L groups decreased sharply within 24 h. In contrast, this decreasing trend in the M and H groups was delayed to 48 h and 60 h respectively. After the hydrolysis/acidogenesis steps, the pH in all groups recovered due to the utilization of volatile fatty acids (VFAs) by methanogens for biogas production (Figure S1). The pH value in all groups decreased with an increasing NaCl concentration, with the final pH of 7.60, 7.56, 7.44 and 7.32 for the blank, L, M and H groups respectively, indicating that an overloading of NaCl could lead to a higher VFAs accumulation and a lower pH value in the AD process.

#### CO_2_ production

**Figure 3:**
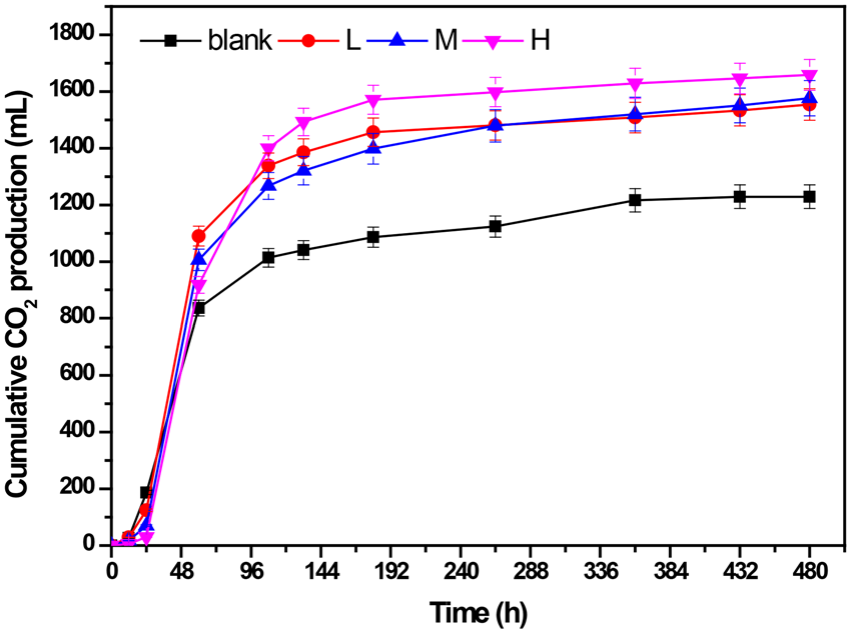
Cumulative CO_2_ productions at different concentrations of NaCl.

The cumulative CO_2_ productions at different concentrations of NaCl were shown in Fig. 3. Overall, the CO_2_ production in all groups was rapidly accumulated within 48 h, where substrates were hydrolyzed and utilized by bacteria. In this step, the L group exhibited the highest CO_2_ production rate, followed by the M group and the H group, while the blank group showed the lowest CO_2_ accumulation rate. However, the CO_2_ production rates in the L, M and blank groups were slowed down afterwards, while the H group maintained a high CO_2_ accumulation rate. As a result, the final cumulative CO_2_ productions for blank, L, M and H group were 1228.7 ± 41.7 mL, 1554.2 ± 55.6 mL, 1576.6 ± 62.4 mL and 1658.7 ± 54.9 mL respectively.

### Overview of the 16S rRNA high-throughput sequencing results

For bacterial communities analysis, 42,855~53,163 clean reads were obtained for each sample with an average length of 412 bp after removing low quality sequences and chimeras. The sequence number of each sample was normalized and 491~530 operational taxonomic units (OTUs) were generated. Analysis of archaeal communities resulted in 44,098~57,074 sequences for each sample with an average length of 382 bp. Richness and diversity of the bacterial and archaeal communities, indicated by OUTs, Chao 1 value and Shannon index, were calculated and listed in Table 1.

**Table 1:** Effective DNA sequences, richness and alpha diversity from bacterial and archaeal communities analysis.

Microbial communities	Samples	Effective reads	OUTs	Chao 1^a^	Shannon index^b^
Bacterial	0 h	42,855	491 ± 9	527 ± 26	4.82 ± 0.02
	B-480 h	53,163	530 ± 11	593 ± 15	5.23 ± 0.16
	H-480 h	47,666	515 ± 18	570 ± 17	4.30 ± 0.21
Archaeal	0 h	51,127	276 ± 7	299 ± 11	4.25 ± 0.04
	B-480 h	44,098	254 ± 11	270 ± 13	3.84 ± 0.37
	H-480 h	57,074	241 ± 17	264 ± 18	3.01 ± 0.25

^a^Chao 1 richness estimator: a higher number represents higher richness.

^b^Shannon index (H): a higher value indicates more diversity.

For the bacterial community, the OUTs and Chao 1 values in both the blank and H groups at 480 h showed no significant difference (*P* > 0.05), but both were higher than those of the original sludge at 0 h (*P* < 0.05). The Shannon index in the blank group significantly increased from 4.82 ± 0.02 to 5.23 ± 0.16 during the AD process, while it decreased to 4.30 ± 0.21 in the H group (*P* < 0.05), demonstrating that the high concentration of NaCl could significantly reduce the sludge diversity. Towards the archaeal community, the richness of both groups was reduced, as reflected by a reduced number of OTUs, Chao 1 (Table 1). Moreover, the blank group showed a much higher Shannon index compared with that of the H group at 480 h (3.84 ± 0.37 vs. 3.01 ± 0.25, *P* < 0.05). This also revealed that the high salinity negatively affected the diversity of the archaeal community. Meanwhile, three significant tests, i.e. Adonis, ANOSIM (analysis of similarity) and MRPP (multi-response permutation procedure), were carried out based on Jaccard distances to analyze the differences of microbial communities of different samples (Table S2). A *P* value less than 0.05 meant a significant difference. The difference analysis indicated that the microbial communities of different samples were significantly different (all *P* < 0.05).

### Microbial community variations at phyla level under different inhibitory conditions

**Figure 4:**
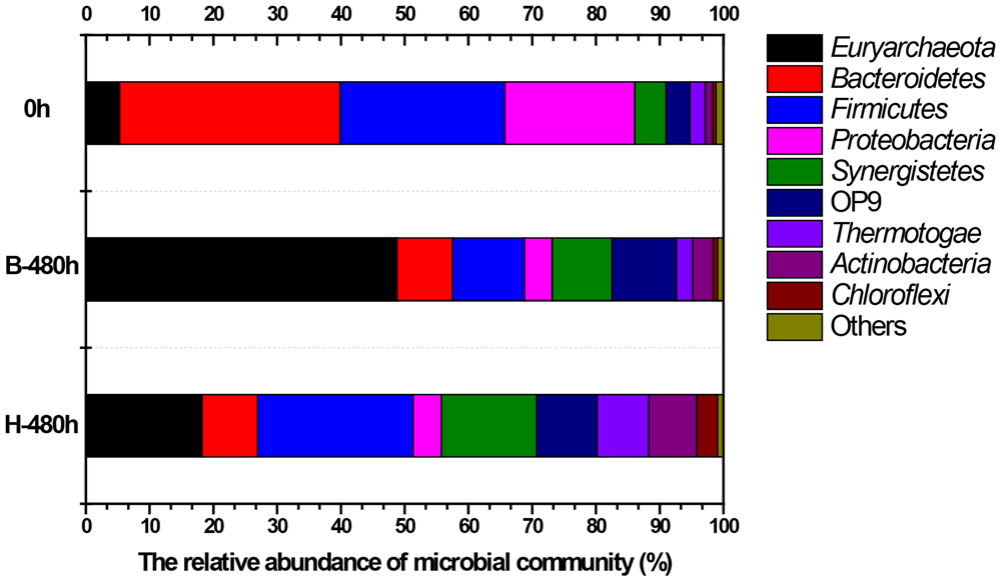
Taxonomic composition of the microbial community at phyla level in each sample. The sequence percentage is above 1% in at least one sample.

The taxonomic compositions of the microbial community at phyla level were shown in Fig. 4. Of the total sequences, less than 2% were not classified at any phylum level. In general, the phylum *Euryarchaeota*, which includes all known methanogens, significantly increased from 5.29% (0 h) to 48.77% (blank group) and 18.26% (H group) after 480-hour operation in an AD process. For bacterial communities, *Bacteroidetes* (34.59%), *Firmicutes* (25.82%) and *Proteobacteria* (20.41%) were the three dominant phyla in the original sludge (0 h). However, a considerable decrease could be observed in both the blank and H groups in abundance of *Bacteroidetes* (8.76% and 8.66%) and *Proteobacteria* (4.33% and 4.45%) during the AD process. *Firmicutes* significantly decreased to 11.29% in the blank group while it remained relatively stable (24.41%) in the H group. In addition, the abundance of *Synergistetes* (9.36% and 14.92%) and OP9 (10.11% and 9.52%) also increased in both the blank and H groups. Comparing the bacterial community changes between the two groups in the late phase, *Firmicutes*, *Thermotogae*, *Actinobacteria* and *Chloroflexi* were more dominant in the H group while *Bacteroidetes*, *Proteobacteria* and OP9 showed no distinct change under high concentrations of NaCl.

### Bacterial community variations at genus level under different inhibitory conditions

**Figure 5:**
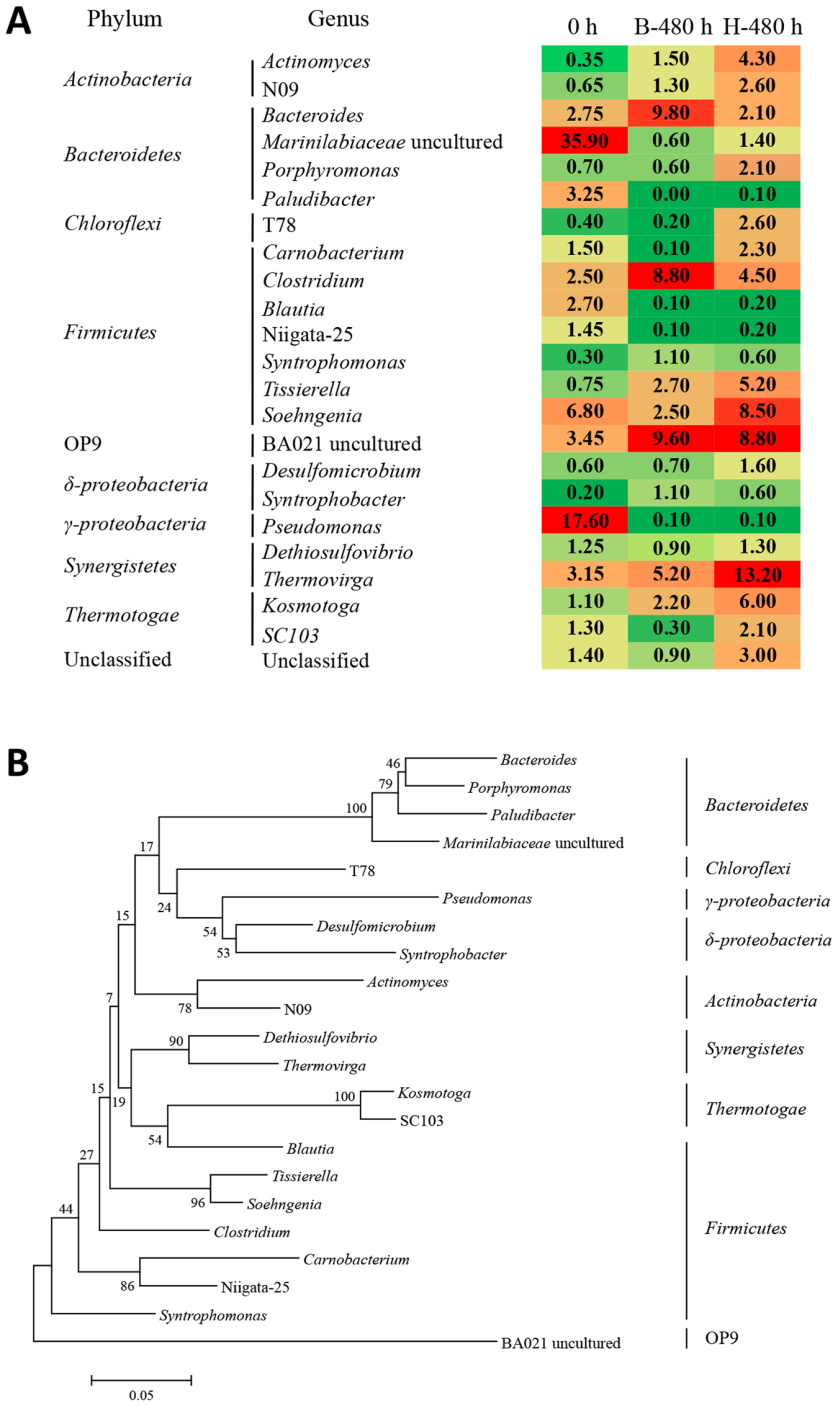
Percentages of the major genera (**A**) in each sample and neighbor-joining phylogenetic tree (**B**) of different genera. The major means sequence percentage is above 1% in any sample. 0 h stands for the samples that were collected immediately after inoculation. B-480 h and H-480 h stand for the samples at 480 h of the blank and H group respectively. A more red intense color corresponds with higher percentages, and deeper green colors indicate the lower percentages. For the phylogenetic tree construction, the neighbor-joining method was used and sequences were aligned using Clustal X 1.8 and MEGA 5.1. The bar represents 5% sequence divergence.

To further reveal the bacteria shift under high salinity inhibition, the bacterial communities of the blank and H groups at genus level were analyzed and the phylogenetic tree showing the phylogenetic identities of different genera was illustrated in Fig. 5. At the genus level, *Marinilabiaceae* uncultured bacterium (35.90%) and *Pseudomonas* (17.60%) were the two primary genera in the original sludge, but were hardly detected after the AD process (Fig. 5A). Other major genera included *Bacteroides* (2.75%), *Paludibacter* (3.25%), *Clostridium* (2.50%), *Blautia* (2.70%), *Soehngenia* (6.80%), BA021 uncultured (3.45%) and *Thermovirga* (3.15%). However, bacterial communities and major genera greatly shifted in the blank and H groups after 480-hour of AD. In the blank group, *Bacteroides* (9.80%), BA021 uncultured (9.60%) and *Clostridium* (8.80%) increased to be the three foremost genera, followed by *Thermovirga* (5.20%), *Tissierella* (2.70%), *Soehngenia* (2.50%) and *Kosmotoga* (2.20%). In the H group, *Thermovirga*, BA021 uncultured and *Soehngenia* were noted to be the most three prevalent genera, with relative abundance of 13.20%, 8.80% and 8.50%, respectively. *Actinomyces* (4.30%), *Clostridium* (4.50%), *Tissierella* (5.20%) and *Kosmotoga* (6.00%) increased to some extent compared with the blank group. In addition, the percentage of *Bacteroides* decreased to 2.10% while other genera affiliated to the same phylum such as *Marinilabiaceae* uncultured bacterium (1.40%) and *Porphyromonas* (2.10%) increased (Fig. 5B).

### Archaeal community variations at genus level under different inhibitory conditions

The relative abundances of the archaeal community at genus level were shown in Table 2. There were only four major genera, *Methanosaeta*, *Methanolinea*, *Methanospirillum*, and *Methanoculleus*, that were predominant in both groups where these genera accounted for more than 99.5% of all archaea. The genus *Metanosaeta* was extraordinary dominant in the original sludge (91.32%), but significantly reduced to 69.82% in the blank group during the AD process. However, this genus showed more dominant in the H group with sequence percentage of 84.59%. Comparatively, the genus *Methanolinea*, which was the second primary genera in the original sludge (6.71%), increased continuously reaching 25.12% and 13.99% in the blank and the H group respectively. In addition, *Methanospirillum* and *Methanoculleus* also showed a modest increase in the blank group with a percentage of 2.74% and 1.46% while they were hardly detected in the H group. It was worth noting that all the major hydrogenotrophic methanogens showed a significantly decrease in response to high salinity stress while the aceticlastic methanogens increased.

**Table 2:** Taxonomic composition of archaeal community at genus level of blank and H groups.

Genus	Functional group	Relative abundance		
		0 h	B-480 h	H-480 h
*Methanosaeta*	Aceticlastic	91.32%	69.82%	84.59%
*Methanolinea*	Hydrogenotrophic	6.71%	25.12%	13.99%
*Methanospirillum*	Hydrogenotrophic	0.77%	2.74%	0.36%
*Methanoculleus*	Hydrogenotrophic	0.58%	1.46%	0.57%
Others	—	0.39%	0.46%	0.18%

## Discussion

Some literature reported that low salinity concentrations were beneficial for the growth methanogens with value of 350 mg Na^+^/L (~0.8 g/L NaCl), while 8~13 g/L NaCl would cause moderate inhibition and values over 20 g/L NaCl would lead to severe impairment^21–23^. Consistent with these studies, the cumulative CH_4_ productions in Fig. 1 showed no obvious difference among the blank, L and M groups (all *P* > 0.05). Moreover, the L group even showed higher production rate before 264 h. The H group however exhibited a strong inhibition with a methane production of 1301 ± 125.7 mL, which was 42.2% lower than that of the blank group.

Two distinct phases, acidogenesis and methanogenesis, could be observed in the pH variation (Fig. 2). During the first phase, i.e. acidogenesis, organic matter was converted to VFAs and CO_2_, thus resulting in an increasing VFA concentration and decreasing pH. Notably, the acidogenesis phase lasted 30 hours in the blank and L groups, while it was extended to 48 and 60 hours in the M and H groups respectively. Ren *et al*. reported that it required some time before microorganisms (mostly bacteria in this phase) became dominant and displayed their functions^24^. In the present study, the higher the NaCl concentration was, the longer the time it required. It should be noted that the pH levels in the four groups did not vary significantly with a minimum value ranging from 6.45 to 6.65 during this phase, despite being extended at high salt concentrations (10–20 g/L NaCl). In addition, VFA accumulation as shown in Figure S1 also supported this conclusion, where the VFA concentrations in the M and H groups were even higher than those of the blank and L groups. However, this higher VFA accumulation should be attributed to the lower VFA assimilation efficiency of methanogens which was inhibited by a high salt concentration. Therefore, it could be concluded that the increasing NaCl concentrations from 0 g/L to 20 g/L might not have a great impact on the substrate consumption by bacteria in the acidogenesis phase. Figure 3 moreover showed that the supplement of additional NaCl (L, M and H groups) did not inhibit but even improve the CO_2_ production compared with that of the blank group in acidogenesis, which might also support this conclusion. During the second phase, i.e. methanogenesis, the VFAs generated during the acidogenesis were used for methane production, thus resulting in a decreasing VFA concentration and increasing pH. However, pH levels varied significantly at high salt concentrations (Fig. 2). The final pH values were only 7.44 and 7.32 in the M and H groups, compared with values of 7.56 and 7.60 in the L and blank groups, indicating that the methanogens might be inhibited, especially in the H group.

**Figure 6:**
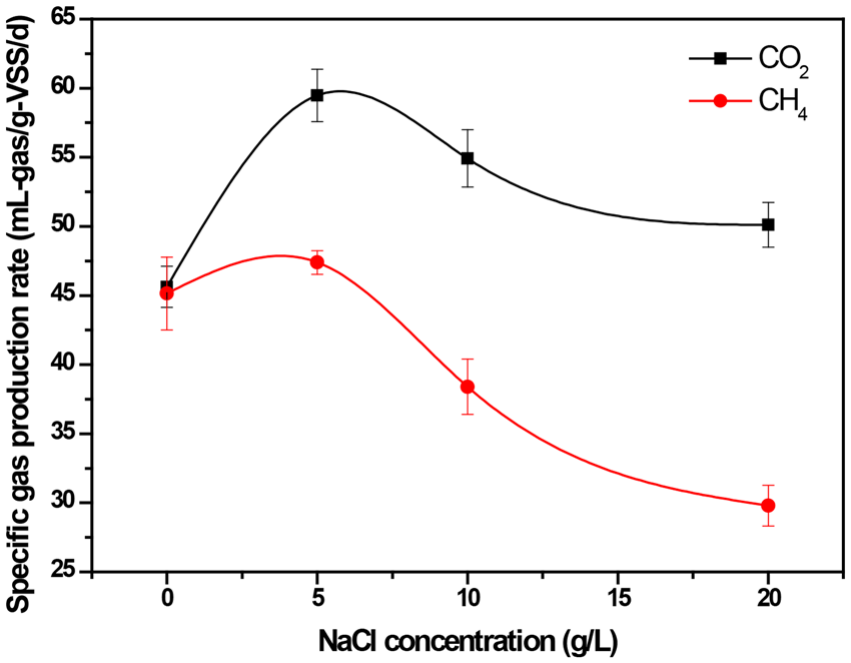
Comparison of specific CH_4_ and CO_2_ generation rates in different NaCl concentrations.

To further quantify the NaCl inhibition on the acidogenic and methanogenic activity, the specific CO_2_ production rate of bacteria obtained in acidogenesis and the specific CH_4_ generation rate of archaea calculated in methanogenesis were compared and shown in Fig. 6. The highest specific CO_2_ (59.47 ± 1.90 mL CO_2_ g^−1^ VSS day^−1^) and CH_4_ production rates (47.39 ± 0.86 mL CH_4_ g^−1^ VSS day^−1^) were obtained at 5 g/L NaCl in the L group, while NaCl concentration exceeding 5 g/L would decrease both the specific CO_2_ and CH_4_ production rates, suggesting that the biogas production could be enhanced by supplying an appropriate salt concentration (5 g/L NaCl in this case). More importantly, although it could lead to a reduction in the specific CO_2_ production rate when the NaCl concentration exceeded 5 g/L, the specific CO_2_ production rate in the H group was even higher than that in the blank group (50.11 ± 1.61 vs. 45.62 ± 1.90 mL CO_2_ g^−1^ VSS day^−1^, *P* < 0.05). This result further proved the above conclusion that the increasing NaCl concentrations did not negatively affect the degrading capability of bacteria in acidogenesis. Instead, it could enhance the acidogenic effect even when NaCl concentration reached 20 g/L. Unlike acidogenesis, the specific CH_4_ generation rate in methanogenesis showed that a significant inhibition occurred when the NaCl concentrations increased from 5 to 20 g/L, which caused a decrease of 37.12% of the specific CH_4_ generation rate (from 47.39 ± 0.86 to 29.80 ± 1.48 mL CH_4_ g^−1^ VSS day^−1^, *P* < 0.05). The results suggested that methanogenesis, rather than acidogenesis, was strongly affected at 20 g/L NaCl in the H group.

The above results showed that a high NaCl concentration could differently affect bacteria and archaea. In order to provide a detailed insight into how the microbial community changes the biogas production under high salinity inhibitory conditions, the samples of the blank and H groups at different periods (0 h and 480 h) were selected for further investigation by Illumina high-throughput analysis. Table 1 illustrated that the bacterial richness of both the blank and H groups showed no difference (*P* > 0.05), while the diversity of the H group was less than the blank group. The result indicated that the high salinity only reduced the bacterial diversity rather than their richness during the AD process. However, the archaeal richness and diversity of the H group was significantly lower than that of the blank group, suggesting that both the richness and diversity of archaea were inhibited by the high NaCl concentration. These results were consistent with the above conclusion that methanogenesis showed more inhibition than acidogenesis in the H group.

Dominant microbial communities of the two groups were notably different (Fig. 4). As commonly found in various AD processes, phyla *Bacteroidetes* (34.59%), *Firmicutes* (25.82%) and *Proteobacteria* (20.41%) dominated in the original sludge (0 h). These bacteria were responsible for biomass degradation and digestion^25, 26^. After 480 h of AD, *Euryarchaeota* exhibited an extremely high abundance (48.78%) while the relative abundance of bacteria such as *Firmicutes*, *Bacteroidetes*, and *Proteobacteria* significantly decreased due to the shift from acidogenesis to methanogenesis. However, the high concentration of NaCl as supplement changed the phyla abundance. *Euryarchaeota* significantly decreased from 48.78% to 18.26%, suggesting that the methanogenesis was severely inhibited. Interestingly, the phyla *Firmicutes*, *Synergistetes*, *Thermotogae* and *Actinobacteria*, which were important bacteria for substrate hydrolysis in acidogenesis, even increased in comparation with the blank group (Fig. 4). This also further indicated that methanogenesis rather than acidogenesis was significantly inhibited in the H group.

Figure 5 illustrated more details about the change of the blank and H group at genus level. For the blank groups, the phylum *Bacteroidetes*, which was the primary dominant bacteria (34.59%) in the original sludge, severely decreased to 8.76% in the AD process. It could be attributed to the significant decrease of *Marinilabiaceae* uncultured bacterium from 35.9% to 0.6% (Fig. 5A). Although it was reported that the family of *Marinilabiaceae* was obligatory anaerobic and saccharolytic bacteria^27, 28^, these bacteria might not be suitable for the AD process and disappeared. However, *Bacteroides*, which was affiliated to the same phylum, was the major in the blank group with a proportion of 9.80%. It was reported that these species could secrete different hydrolyzing enzymes such as cellulase, amylase, protease and lipase^29^, indicating its importance for the depolymerisation of organic matter in the acidogenesis phase. *Clostridium* was also found dominant in the blank group (8.80%). These species were widely distributed in various anaerobic systems and played an important role in the acidogenesis/acetogenesis stage^7, 30^. Our previous work demonstrated that these acid-generating species such as *Bacteroides* sp. and *Clostridium* sp. could reduce the pH value and further affect the bacterial community, leading to an increase of the adaptive species and a decrease of the non- adaptive species^31^.

The microbial community in the H group also shifted and a greater evenness of species was observed (Fig. 5A). For example, *Bacteroides* decreased from 9.8% to 2.1%, while other genera such as *Actinomyces* (4.30%), N09 (2.60%), and *Porphyromonas* (2.10%) increased and displayed similar hydrolytic abilities^32, 33^. The percentage of *Clostridium* decreased from 8.80% to 4.50%, whereas two major genera, *Tissierella* and *Soehngenia*, which belong to the same family *Tissierellaceae*, were found to be more abundant than *Clostridium* in the H group within this phylum (Fig. 5B). These two genera were also reported to be functionally important in acidogenesis^34, 35^. *Thermovirga* was observed to be the most prevalent genera in the H group at the late phase, with relative abundance of 13.2%. *Thermovirga* was reported to have a high tolerance to high salinity^36^ and dispalyed a high hydrolysis ability^37^. As described above, the H group was not inhibited in acidogenesis, in which VFAs accumulation and CO_2_ production were similar to the blank group (Fig. 3 and Figure S1). This further revealed that the microbial community changed in high salinity condition to maintain the hydrolysis capacity.

It should be noticed that both the blank and H groups harbored a significantly high percentage of BA021 uncultured bacterium (9.6% and 8.8%, respectively), which was assigned to candidate phylum OP9 (now termed as phylum *Atribacteria*). With the help of culture-independent technologies, these bacteria were frequently detected in anaerobic digesters, petroleum reservoirs, and deep marine sediment^38^, indicating that these bacteria might be tolerant to a high concentration of NaCl. Moreover, a recent study reported that the phylum OP9 was abundant in high-methane conditions, and might play a key role in regulating both the production of methane and the diversity of methane producers^39^.

Taken together, although a significantly bacterial community shift could be observed between the H group and the blank group and many bacteria were inhibited under high salinity conditions, a variety of halotolerant bacteria that exhibited similar hydrolytic and acidogenetic abilities adapted to the high salt concentrations and even became dominant. Therefore, in line with the above conclusion, the increasing NaCl concentrations might not have a great impact on the substrate degradation. As previously stated, the decrease in methane production might largely relate to the inhibition of the archaeal community.

Unlike the bacterial community, the archaeal compositions in the both groups were quite simple with only four major genera representing more than 99.5% of all archaea (Table 2). Generally, the relative abundance of phylum *Euryarchaeota* decreasing from 48.77% (blank group) to 18.26% (H group) suggested the significant inhibition in response to high salinity stress (Fig. 4). Detailed archaeal community analysis at genus level showed that the aceticlastic methanogen *Methanosaeta* was the most important dominant species in the both groups, which was consistent with our previous result^31^. *Methanosaeta* was reported to be the predominant methane producer on earth^40^ and showed a high affinity and low minimum threshold for acetate^41, 42^. These species could consume acetate for CH_4_ production, resulting in an increase of pH and decrease of VFAs (Fig. 2 and Figure S1). It was worth noting that the relative abundance of *Methanosaeta* increased from 69.82% to 84.59% with elevated NaCl concentrations (Table 2), indicating that these species might not be affected significantly in the H group.

Kim *et al*. reported that the methanogen population would shift from acetoclastic methanogens to hydrogenotrophic methanogens with the release of VFAs^19^. Consistently in the blank group, the hydrogenotrophic methanogens such as *Methanolinea*, *Methanospirillum* and *Methanoculleus* significantly increased with the relative abundance of 25.12%, 2.74% and 1.46% respectively (Table 2). These hydrogenotrophic genera were capable of producing CH_4_ through the reduction of CO_2_ with H_2_ and could use formate and alcohols as alternative electron donors^4, 43^. However, it seemed that the shift from acetoclastic methanogens to hydrogenotrophic methanogens was significantly inhibited in the H group due to the high concentration of NaCl. The major hydrogenotrophic genus *Methanolinea* decreased dramatically to 13.99% while the other two genera were hardly detected (Table 2). According to our experiment data in Fig. 3, the cumulative CO_2_ production of the H group was 34.99% higher than that of the blank group, whereas this increase should be largely attributed to the inhibition of hydrogenotrophic methanogens.

To verify the above conclusion, the final CH_4_ and CO_2_ concentrations of the blank and H groups were further analyzed and shown in Figure S2. As mentioned above, hydrogenotrophic methanogens could utilize H_2_ and CO_2_ as substrate to produce CH_4_^44^. Therefore, a higher CO_2_ and lower CH_4_ concentration would be expected if hydrogenotrophic methanogens were inhibited. As expected, a significant decrease in CH_4_ concentration (from 61.08 ± 1.47 to 55.57 ± 0.43, *P* < 0.05) and increase in CO_2_ concentration (11.79 ± 0.66 vs. 14.21 ± 0.18, *P* < 0.05) could be observed in the H group than in the blank group. Hence, the high salinity might be less toxic to aceticlastic methanogens than to hydrogenotrophic methanogens.

In summary, the impact of increasing NaCl concentrations on the biogas production was systematically investigated and the Illumina high-throughput sequencing method was employed to reveal the microbial community variations in response to high salinity stress. The increasing NaCl concentrations from 0 g/L to 20 g/L did not negatively affect the activity of bacteria during acidogenesis but even promoted it, while the methanogenic activity in methanogenesis was strongly inhibited. Microbial communities between the blank and H groups were significantly different. *Bacteroides*, *Clostridium* and BA021 uncultured were the major genera in the blank group. Nevertheless, these species were inhibited to some extent in the H group while some halotolerant bacteria, such as *Thermovirga*, *Soehngenia* and *Actinomyces*, were dominant and displayed similar hydrolytic and/or acidogenetic abilities. The archaeal community compositions were quite simple and were more easily affected by high salinity. Hydrogenotrophic methanogens showed a lower resistance to high salinity than aceticlastic methanogens.

## Materials and Methods

### Inoculums and anaerobic digestion

The seed sludge was obtained from a 100 m^3^ food waste anaerobic digester in the Changping district, Beijing, and had been acclimated over one year for FW treatment. The seed sludge was kept for 5~8 days at 35 °C to consume the residual organic matter before inoculation.

The batch anaerobic digestion was operated using 1 L glass digesters with total solids (TS) of 5 wt% for anaerobic digestion at mesophilic temperature (35 ± 1 °C). To avoid the influence of the high fluctuating compositions of FW on the AD process, starch and peptone were used instead of FW. The inoculum and substrates were fully mixed before being added to the digesters. After inoculation, each digester was flushed for 10 min (300 mL/min) with argon gas to provide anaerobic condition. Prior to this study, the medium was optimized to be 6 g/L starch and 1.22 g/L peptone (C/N = 15). Four different groups were examined in triplicates, where NaCl was supplemented in increasing concentrations of 0 g/L (blank group), 5 g/L (L group), 10 g/L (M group) and 20 g/L (H group), respectively. All the digesters were operated under identical conditions. All the reagents used in this study were analytically pure and were purchased from Xilong Scientific Co., Ltd, Beijing, China.

### DNA extraction and pyrosequencing

Sludge samples of the original and final stages of the blank and H groups were collected and stored at −80 °C before use. The total genomic DNA was extracted using Soil DNA extraction kit (OMEGA, Georgia, United States). The DNA concentration was determined by Nano drop (NanoDrop Technologies, Inc, Wilmington, United States). Prepared genomic DNA samples were sent to Novegene (Beijing, China) for shotgun library construction using an Illumina Hiseq2500 platform. The raw sequences were joined and treated according to Hu *et al*.^45^. Quantitative Insights Into Microbial Ecology (QIIME) 1.7.0 was used to sift the raw reads, the resulting high quality sequences were clustered into operational taxonomic units (OTUs) at the 97% sequence similarity threshold by Uclust clustering. The phylogenetic tree was constructed by MEGA version 5.1, using the neighbor-joining method.

### Analytical methods

The total solid (TS), volatile solid (VS) and volatile suspended solid (VSS) were measured according to the standard methods^46^. The pH was monitored by an ion meter (MP 523 pH/ISE meter, San-Xin Instrumentation, Inc, Shanghai, China). Biogas was collected by water displacement method. Biogas and VFAs concentrations were examined by gas chromatography (GC-2014C, Shimadzu, Kyoto, Japan) using a thermal conductivity detector (TCD) and hydrogen flame ionization detector (FID) respectively, as detailed in a previous study^47^.

### Statistical analysis

Cluster analysis was performed to evaluate the overall differences in microbial community structure based on Jaccard distances. Dissimilarity tests (i.e. MRPP, ANOSIM and Adonis) were performed to determine the significance of differences between the microbial community compositions in R v.3.3.2 using the ‘vegan’ package^48^. Data were analyzed using SigmaStat 3.5. The one-way ANOVA were used to test the significance of differences between groups, and *P* < 0.05 was considered as significant.

## Electronic supplementary material

Supplementary Information
