# Supplementary material for: Exploration of the relationship between biogas production and microbial community under high salinity conditions
Source: Sci Rep. 2017 Apr 25;7:1149. doi: 10.1038/s41598-017-01298-y (PMC5430677; doi:10.1038/s41598-017-01298-y)
Supplement: Supplementary file 1 — Supplementary Information [file 41598_2017_1298_MOESM1_ESM.doc]

**Supplementary Information**

**Exploration of the relationship between biogas production and microbial community under high salinity conditions**

Shaojie Wang, Xiaocong Hou, Haijia Su*

Beijing Advanced Innovation Center for Soft Matter Science and Engineering, Beijing Key Laboratory of Bioprocess, Beijing University of Chemical Technology, Beijing 100029, People’s Republic of China.

*Corresponding author: [suhj@mail.buct.edu.cn](mailto:suhj@mail.buct.edu.cn)

**Table S1 The biogas and methane yield at different conditions.** The C/N radio was kept at 15 in all the experiments.

| Starch concentrations (g/L) | 6 | 9 | 12 |
| --- | --- | --- | --- |
| Biogas yield (mL∙/g-VS) | 746.83 | 510.56 | 580.92 |
| Methane yield (mL∙/g-VS) | 305.91 | 236.90 | 245.71 |

**Table S2 Significance tests of microbial communities based on Jaccard distance.** Adonis, permutational multivariate analysis of variance with the Adonis function. ANOSIM, analysis of similarity. MRPP, multi-response permutation procedure. *P* values less than 0.05 were considered significant.

| Groups | Adonis | ANOSIM | MRPP |
| --- | --- | --- | --- |
|  | *P* | *P* | *P* |
| 0 h vs. B-480 h | 0.001 | 0.009 | 0.002 |
| 0 h vs. H-480 h | 0.001 | 0.012 | 0.002 |
| B-480 h vs. H-480 h | 0.001 | 0.007 | 0.001 |


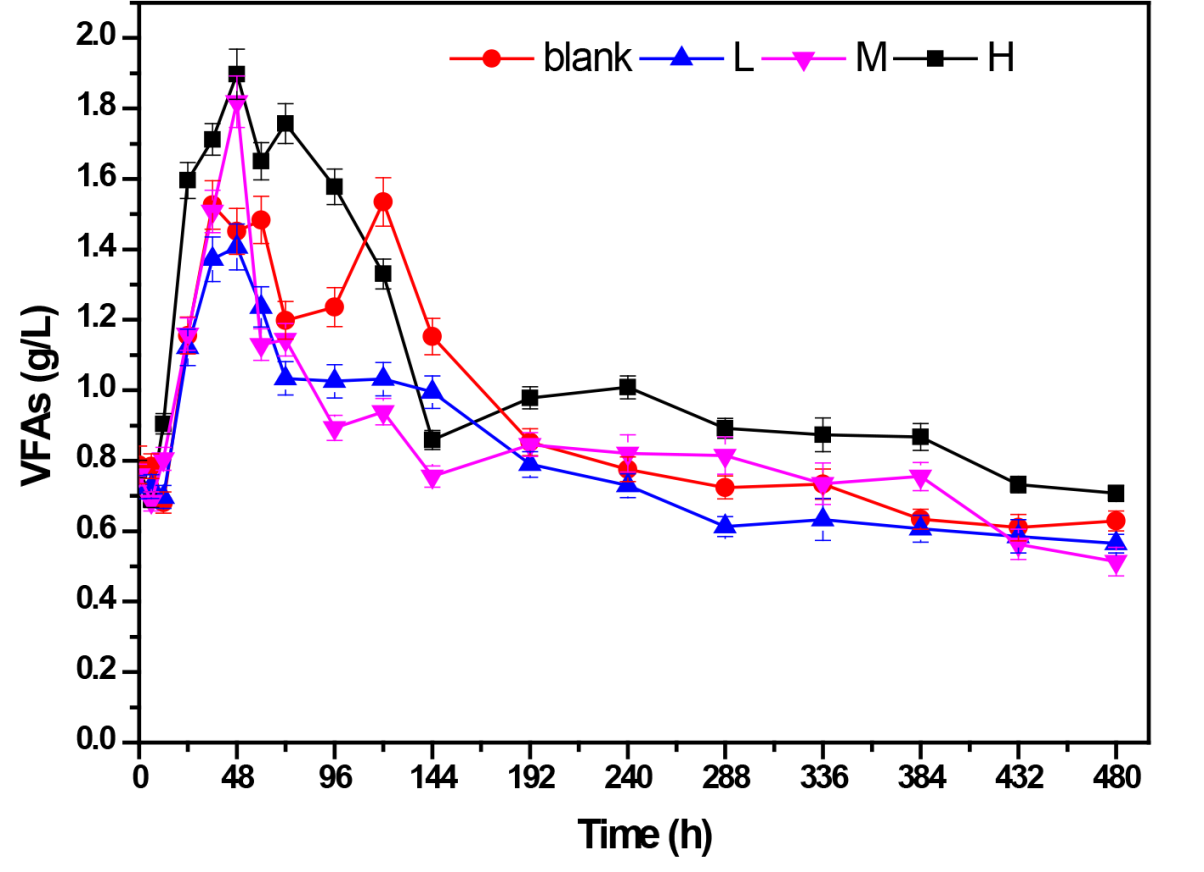


**Figure S1**. **VFAs variations at different concentration of NaCl.** Different concentrations of NaCl were added at the blank group (0 g/L), L group (5 g/L), M group (10 g/L) and H group (20 g/L). Average values and error bars were calculated from three replicates.


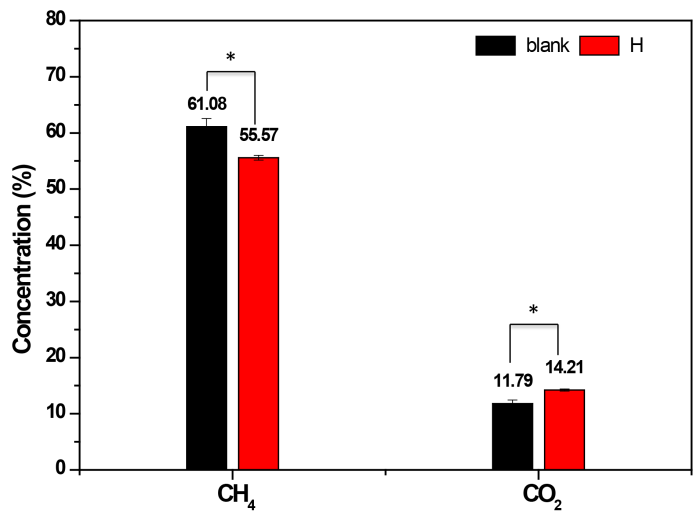


**Figure S2. Comparison of final CH_4_ and CO_2_ concentrations between the blank and H groups.** Different concentrations of NaCl were added at the blank group (0 g/L) and H group (20 g/L). Error bars represent SEM; *P* < 0.05 was considered significant and is indicated by *.
